# Melt Quenching Process for the Synthesis of Na_5_MSi_4_O_12_-Type Compounds, Sodium-Ion Conducting Solid Electrolytes

**DOI:** 10.1021/acsomega.6c08181

**Published:** 2026-09-02

**Authors:** Anna Michalak, Urvashi Urvashi, M. Anji Reddy

**Affiliations:** IMPACT Energy Storage Laboratory, Faculty of Science and Engineering, 7759 Swansea University, Swansea SA1 8EN, U.K.

## Abstract

Na_5_MSi_4_O_12_-type compounds are emerging as viable solid electrolytes for solid-state sodium batteries because of their high ionic conductivity and excellent stability. However, Na_5_MSi_4_O_12_-type compounds are often prepared by traditional solid-state synthesis, which involves extensive ball-milling, long sintering times, and multiple processing steps to produce high-purity materials. This leads to long preparation times and energy inefficiency. Here, we show that glasses of Na_5_MSi_4_O_12_ (M = Y, Dy, Gd, and Sm) can be synthesized via a simple melt-quenching process. Subsequent annealing of these glasses at respective temperatures resulted in highly conductive glass ceramics. The structure and properties of these glasses and glass-ceramics were studied using simultaneous thermal analysis, XRD, SEM, FTIR, Raman, and EIS. This article presents an energy-efficient, simple, and alternative approach to the synthesis of promising Na_5_MSi_4_O_12_-type solid electrolytes for solid-state sodium-ion batteries.

## Graphical abstract

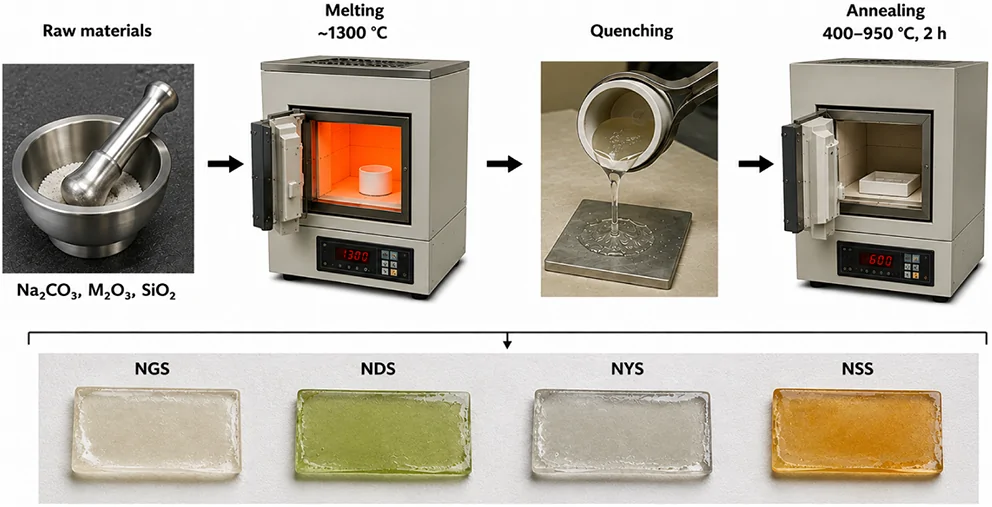

## Introduction

Among alternative battery technologies to lithium-ion batteries (LIBs), sodium-ion batteries (SIBs) are promising owing to their low cost, sustainability, and offer many other advantages.^1^ However, high-performance solid electrolytes (SEs) are needed to replace current liquid electrolytes and make SIBs safer and more energy-dense. Na_5_MSi_4_O_12_-type (NMS) SEs are suitable for this purpose, as their ionic conductivity and electrochemical stability are on par with liquid electrolytes.^2^ However, these compounds are often prepared by traditional solid-state synthesis, which involves extensive ball-milling, long sintering times, and multiple processing steps to produce high-purity materials. This leads to lengthy preparation times and energy inefficiency.^3^ To overcome the limitations of traditional solid-state synthesis, alternative, more energy-efficient methods must be adopted and developed to enable large-scale synthesis. One such method is melt quenching (MQ), which involves rapid cooling of a molten phase to form an amorphous glass, followed by subsequent controlled crystallization to produce polycrystalline ceramics. Materials prepared this way can be shaped into thinner components by pouring the melts directly into molds, which could be advantageous for solid-state battery fabrication by minimizing series resistance in the electrochemical cells.

However, synthesizing highly conductive Na^+^ oxide-based conductors via the melt-quenching process presents several challenges. For example, the starting powders of BASE (sodium β-alumina) materials are hygroscopic; therefore, their exposure to atmospheric conditions should be minimized.^4^ In addition, NASICON materials require high melting temperatures and exhibit uneven melting due to their high ZrO_2_ content. Efforts to synthesize NASICON-like glass ceramics with reduced ZrO_2_ content have generally resulted in materials with low ionic conductivity.^5–7^ However, melt-quenching has been successfully used to synthesize NMS-type solid-state compounds. Banks et al. were the first to explore this approach, crystallizing the NMS materials from glasses containing rare-earth elements (Er, Y, Gd, and Sm) by melting them between 1300 and 1350 °C and holding for 2 to 3 h, followed by crystallization at 890–1000 °C. The resulting glass-ceramics exhibited high sodium conductivity in the range of 10^–2^ S cm^–1^ at temperatures above 200 °C,^8^ demonstrating that highly conductive NMS electrolytes could be produced through glass-forming techniques without significantly compromising their fast-ion conducting properties. However, the authors provided no information on glass transition temperatures.

Subsequent studies by Okura, Yamashita, and co-workers expanded the compositional flexibility of NMS-based glass ceramics by incorporating phosphate (P_2_O_5_) species into the silicate framework. The development of Na_2_O-R_2_O_3_–P_2_O_5_–SiO_2_ (Narpsio) glass systems via a glass-crystallization route enabled precise control of crystal chemistry by modifying the parent glass composition, providing important insights into structure–property relationships in crystallized N5-type Na_5_MSi_4_O_12_ conductors. Using this approach, a wide range of rare-earth cations (Sc, In, Y, La, Nd, Sm, Eu, Gd, Dy, Er, and Yb) were successfully incorporated on the R site, while substitutions involving B, Al, P, Ti, V, Ga, Ge, Mo, and Te were introduced on the Si site.^9–13^ These studies demonstrated that melt quenching offers exceptional compositional flexibility and enables systematic tuning of sodium-ion conduction pathways through targeted cation substitution, thereby establishing melt quenching and glass crystallization as a versatile route for developing new N5-type solid electrolytes.

Recent studies of oxide-based solid electrolytes have further demonstrated that melt-quenching, together with compositional engineering, is an effective strategy for enhancing ionic transport. In NASICON-type Na_3_Zr_2_Si_2_PO_12_,^14,15^ LiTi_2_(PO_4_)_3_,^16^ and Li_1.3_Al_0.3_Ti_1.7_(PO_4_)_3_ ^17,18^ solid electrolytes, improvements in ionic conductivity have been achieved through aliovalent cation substitution, which modifies the crystal structure, widens ion migration pathways, and improves the microstructure after crystallization. These studies highlight continued interest in developing energy-efficient processing routes and structure–property relationships for oxide solid electrolytes, providing further motivation to investigate melt-quenched Na_5_MSi_4_O_12_ glass-ceramics as promising sodium-ion-conducting materials.

Within the NMS family itself, further work continues to demonstrate the versatility of this framework: La doping of Na_5_YSi_4_O_12_ has been shown to improve grain-boundary ionic conductivity,^19^ nonstoichiometry-induced amorphous grain boundaries have been used to suppress dendrite formation in Na_5_SmSi_4_O_12_,^20^ and grain-boundary electronic insulation strategies have recently been reported for related sodium rare-earth silicates,^21^ underscoring sustained interest in optimizing NMS-type electrolytes for practical solid-state sodium batteries.

More recently, Schilm et al. investigated the sintering temperature of glasses produced by the melt-quenching process in NMS (M = Y, Gd, and Sm).^22,23^ The glasses were prepared by melting at 1350 °C, and a maximum ionic conductivity of 1.82 × 10^–3^ S cm^–1^ at 20 °C was observed for NYS.^22^ Collectively, these studies demonstrate that melt-quenching is an effective route for producing highly conductive NMS-based solid electrolytes, offering advantages in processing flexibility, reduced processing times, and potential scalability. In this report, we investigated the glasses and glass ceramics of Na_5_MSi_4_O_12_ (M = Y, Dy, Gd, and Sm) in detail, synthesized via a melt-quenching process followed by subsequent crystallization. We further investigated and reported the structure and properties of NMS-type glasses and their glass ceramics.

## Experimental Section

All starting materials were reagent grade and commercially available. Sodium carbonate (Na_2_CO_3_, 99.5%, Alfa Aesar), gadolinium oxide (Gd_2_O_3_, 99.9%, Thermo Scientific Chemicals), yttrium oxide (Y_2_O_3_, 99.9%, Thermo Scientific Chemicals), dysprosium oxide (Dy_2_O_3_, 99.9%, Thermo Scientific Chemicals), samarium oxide (Sm_2_O_3_, 99.9%, Thermo Scientific Chemicals), and silicon dioxide (SiO_2_, 99.5%, Thermo Scientific Chemicals) were purchased from Thermo Fisher Scientific, United Kingdom.

Phase composition of NGS, NYS, NDS, and NSS was characterized via XRD using a Bruker Discovery 8 diffractometer with Cu–Kα radiation (40 kV; 40 mA). Scans were recorded between 10 and 50°. The XRD patterns were analyzed by DIFFRAC.EVA software and X’Pert HighScore software. The microstructure of the sintered solid electrolyte pellets was examined using the scanning electron microscope Zeiss Evo LS25 SEM. Density measurements of the sintered NYS, NDS, and NSS pellets were performed according to the Archimedean principle by suspending the samples in air and distilled water using a Kern EMB-V balance and density determination kit and measuring the resulting displacement. DSC/TGA tests were carried out using a Netzsch STA 449F1 Jupiter Simultaneous Thermal Analysis instrument. All measurements were carried out from 30 to 1300 °C at a heating rate of 1 °C min^–1^ (unless specified). The TG and DSC data were analyzed by Netzsch Proteus software. FTIR spectroscopy measurements were carried out using a PerkinElmer Spectrum Two FT-IR spectrometer and recorded with PerkinElmer Spectrum IR software in the 1800– 400 cm^–1^ range.

Electrochemical Impedance Spectroscopy (EIS) was performed using a Gamry 1010E potentiostat. The test cells were placed in an incubator (Neware, MHW-25) to adjust the temperature during the measurements. Before the experiment, gold (Au) was sputtered as an ion-blocking electrode on either side of the shaped NMS ceramic piece using an Agar sputter coater. EIS was performed over an AC frequency range from 1 Hz to 2 MHz with an alternating voltage amplitude of 10 mV. The resistance was calculated by extrapolating the curve to the *x* axis. The temperature-dependent impedance was measured from RT to 120 °C to determine the activation energy (*E*_a_). *E*_a_ can be calculated from the following equation:

$$\sigma =A\;\exp\left(-\frac{E_{a}}{kT}\right)$$

where σ is the conductivity (S cm^–1^), *A* is the pre-exponential factor (S cm^–1^), *E*_a_ is the activation energy (eV), *k* is the Boltzmann constant (8.617 × 10^–5^ eV K^–1^), and *T* is the temperature (K).

## Results and Discussion

### Synthesis and Properties of NMS-based glasses

The dried and mixed NMS precursors were heated to 750 °C for 2 h in a muffle furnace to decompose and release CO_2_. The mixture was then melted at 1300 °C and kept at that temperature for 1 h. The melts were quickly quenched in air and removed from the Pt crucibles after cooling to RT. No air bubbles were observed in the fused glasses. The Na_5_GdSi_4_O_12_ (NGS), Na_5_YSi_4_O_12_ (NYS), Na_5_DySi_4_O_12_ (NDS), and Na_5_SmSi_4_O_12_ (NSS) have different melting temperatures ranging from 1000 to 1200 °C. To ensure even melting, all the compounds were heated to 1300 °C for 1 h. We also limited the melting time to 1 h to minimize potential Na evaporation. The glass pieces were annealed in a muffle furnace at 400 °C for 2 h to relieve any thermal stresses and prevent cracking. The heating rate was controlled at 200 °C/hour. Figure S1 shows digital images of the glass pieces. The NYS and NGS glass pieces were colorless and transparent; NDS had a pale-yellow tint, and NSS displayed a yellow-orange hue. XRD patterns of the glass powders are presented in Figure 1. The absence of any crystalline peaks confirms the amorphous nature of the synthesized NMS glasses. The glasses were then cut into standard rectangular shapes using a glass cutter and polished with sandpaper. For NMS crystallization, the shaped glass pieces were heated in a muffle furnace to specific temperatures. Exact heating conditions are tabulated in Table S1, as they vary across NMS compounds.

**Figure 1:**
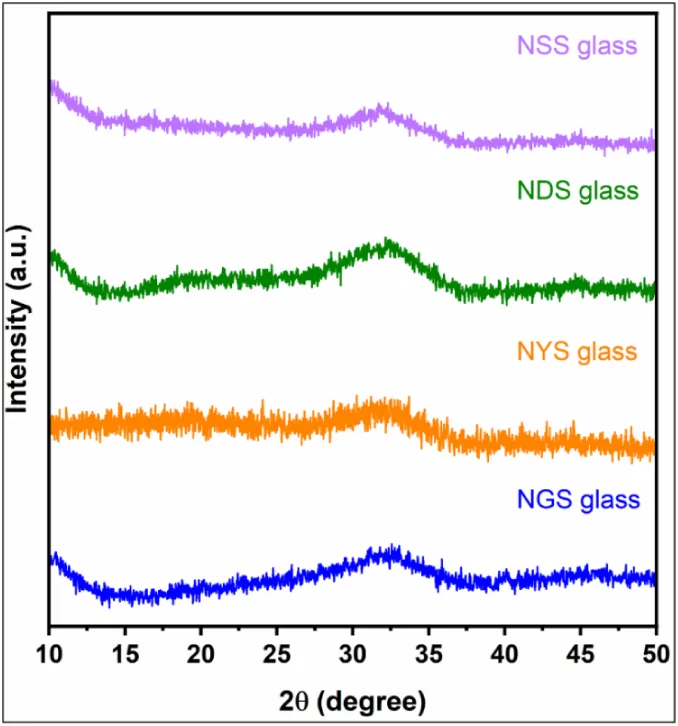
XRD patterns of NMS glass powders.

To examine the thermal behavior of NMS glasses, the glass samples were crushed into powders and analyzed using simultaneous thermal analysis (STA). All measurements were performed from 30 to 1300 °C at a heating rate of 1 °C/min. The glass transition temperature (*T*_g_), crystallization point (*T*_cp_), and melting point (*T*_mp_) of the as-quenched glasses were determined from their corresponding differential scanning calorimetry (DSC) curves using NETZSCH Proteus Thermal Analysis software. Figure 2a presents thermal analysis results for NGS glass. The *T*_g_ of NGS glass is 587 °C. The exothermic peak at 757 °C corresponds to the crystallization peak, which begins at around 687 °C. The endothermic peak at 1077 °C is due to NGS melting. The *T*_g_ of NYS was observed at approximately 616 °C (Figure 2b), the highest among the studied NMS glasses. The exothermic peak observed at 736 °C corresponds to crystallization of NYS. Similar to NGS, the sharp endotherm at 1181 °C corresponds to NYS melting. The *T*_g_ of NDS glass was observed at around 591 °C (Figure 2c). A strong exothermic peak at 736 °C marked the crystallization peak, the same value observed for NYS glass. The melting point of NDS glass was observed at 1166 °C. The *T*_g_ of NSS glass is 557 °C, the lowest among the studied NMS phases (Figure 2d). The *T*_cp_ was observed at 699 °C. The melting point peak of NSS glass was observed at 1088 °C. These results are also tabulated and presented in Table S1.

**Figure 2:**
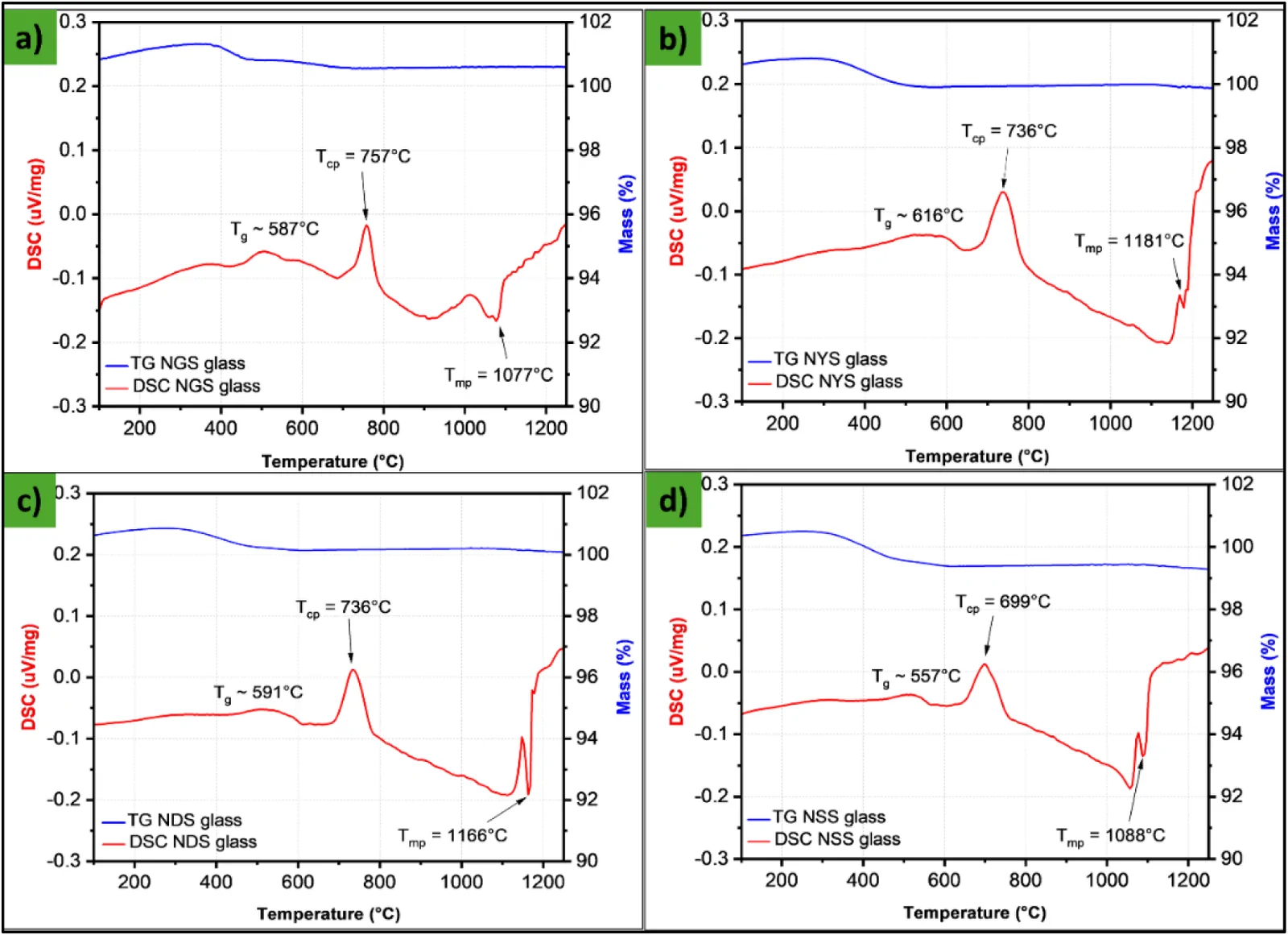
STA of NMS-type glass powders. Measurements were performed from 30 to 1300 °C at a heating rate of 1 °C/min: (a) NGS, (b) NYS, (c) NDS, and (d) NSS. The glass transition temperature (*T*_g_), crystallization point (*T*_cp_), and melting point (*T*_mp_) of the as-quenched glasses were determined from their corresponding differential scanning calorimetry (DSC) curves using NETZSCH Proteus Thermal Analysis software.

Thermal data on NMS glasses are scarce in the literature, with most available data reported on sodium–yttrium–silicate systems.^23–25^ The measured glass transition temperatures for NYS in this study are consistent with previously reported values, which range from 549 to 626 °C, depending on the exact glass composition. However, the crystallization temperatures measured in this study are lower than those previously reported, which ranged from 775 to 876 °C.^24,25^ In another study, NYS glass crystallized at approximately 700 °C,^23^ which is closer to the values obtained in this work.

### Ionic conductivity of NGS glass

We determined the ionic conductivity of the NGS glass through EIS. Before the measurement, the shaped NGS glass sample was coated with a thin layer of gold to create ion-blocking electrodes. Figure S2 shows the impedance spectra for the NGS glass, measured at RT, 200 °C, and 300 °C, presented as Nyquist plots. At RT, the Nyquist plot showed only a straight line. This indicates that the impedance of the NGS glass sample at RT is exceedingly high, making the characteristic semicircle feature of the Nyquist plot undetectable. However, as the temperature increased to 200 °C, a semicircle became more prominent, indicating a significant reduction in the sample’s impedance. This reduction can be attributed to enhanced ionic mobility within the glass matrix as the temperature increases. At 200 °C, the impedance was approximately 27,500 kΩ. Further increasing the temperature to 300 °C reduced the resistance to approximately 7 kΩ. By extrapolating the imaginary component of the complex impedance to its intercept with the real component (*x* axis), the sample’s resistance was determined, enabling the calculation of ionic conductivity. At 200 °C, the ionic conductivity was calculated as 1.48 × 10^–4^ S cm^–1^, increasing to 5.71 × 10^–4^ S cm^–1^ at 300 °C. This ionic conductivity is 1 order of magnitude higher at 200 °C than the values reported by Banks, and of the same order of magnitude at 300 °C. As the ionic conductivity of NGS glass is not significant at RT, no further ionic conductivity measurements were performed on other NMS glasses.

The low ionic conductivity of sodium silicate glasses is expected. In their crystalline form, NMS-type sodium silicates exhibit an ordered 3D structure that facilitates the rapid ion migration. However, in the disordered, amorphous state, the absence of an organized framework impedes Na^+^-ion transport, leading to a significantly lower ionic conductivity. Introducing crystalline phases through controlled thermal treatment substantially enhances ionic conduction by providing defined pathways for the ion migration. Upon heating and crystallization, the ions arrange in an ordered structure, creating well-defined channels within a 3D network that enable rapid sodium ion transport and much higher ionic conductivity in the crystallized NGS solid electrolyte.

### Thermal properties, crystallization and structure of Na_5_GdSi_4_O_12_, Na_5_YSi_4_O_12_, Na_5_DySi_4_O_12_ and Na_5_SmSi_4_O_12_ glass ceramics

The glass ceramics were also investigated by STA. Peaks associated with the crystallization process are absent from the DSC curves of the glass ceramics, confirming that the glass phase has successfully transformed into a glass ceramic material (Figure 3). All samples displayed a broad endothermic peak between approximately 600 and 1100 °C. This behavior is consistent with observations from samples prepared via solid-state synthesis and is attributed to the continuous particle growth. The melting point of the NGS glass ceramic was observed at 1132 °C, which is relatively higher than that of the NGS parent glass at 1077 °C. The melting point of the NYS sample was measured at 1190 °C, slightly higher than that of its glass counterpart, which melted at 1181 °C. The melting point of NDS glass ceramic is 1166 °C, matching that of the glass phase. Similarly, the NSS sample exhibited a melting point of 1088 °C, which remained unchanged from its glass phase. The melting points of NGS and NSS are very close to those previously reported (1139 °C for NGS and 1082 °C for NSS) for the N5 phase by the MQ method, while the melting point of NYS is about 20 °C lower than the reported value (1213 °C). No studies reported the melting point of NDS prepared by MQ synthesis. TG analysis of all samples showed only minor mass losses, approximately 1%, attributed to the evaporation of adsorbed water on the sample surfaces. These minimal mass losses confirm the thermal stability of the solid electrolytes under the experimental conditions.

**Figure 3:**
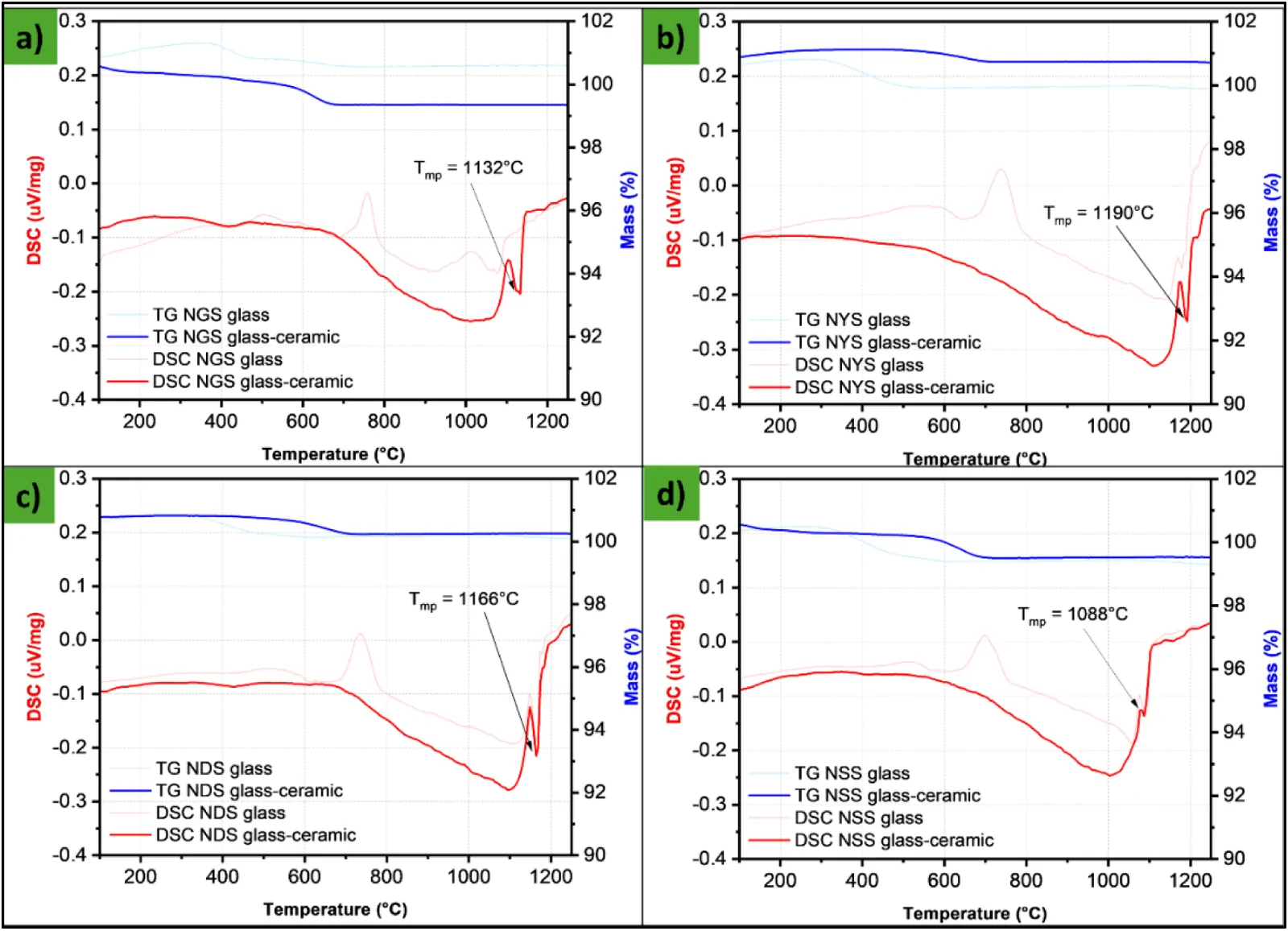
STA of NMS-type glass ceramic powders: (a) NGS, (b) NYS, (c) NDS, and (d) NSS.

Crushing the glass via ball milling, pressing the resulting powders into pellets, and sintering for ionic conductivity measurements led to the formation of the NMS compound with significant impurity peaks (Figure S3). This could be due to partial decomposition of the amorphous N5 phase during ball milling. Therefore, we advise using direct sintering of the glassy amorphous N5 phase to obtain highly conducting glass ceramics. Further, we employed a two-step heat-treatment process to crystallize the bulk glass samples (to avoid potential decomposition of the glass phase). In the first step, the samples were heated at *T*_g_ + 30 °C. This temperature was selected to initiate crystal nucleation within the glass matrix. Above the glass transition temperature, grains nucleate, and crystallization begins. To promote crystallization and to avoid mechanical cracking, a slow heating rate of 75 °C/h was then applied. The second step involved heating the samples to 1050 °C for NGS, NYS, and NDS and to 950 °C for NSS to allow the formation of fully crystallized N5 phases. All samples were held at their respective sintering temperature for 4 h. Following this heat treatment, the samples were quenched in air to preserve the formation of the conductive N5-phase. Please note that gradual cooling led to the formation of impure phases, as reported in our previous paper;^26^ therefore, we opted for quenching. As a result of the sintering process, the initially transparent glass transformed into opaque, white-yellowish samples (digital images are shown in Figure S4).

Figure 4 presents the XRD patterns of NMS phases crystallized from parent glasses obtained by melt quenching. The XRD patterns confirm that the glasses were successfully transformed into crystalline materials after 4 h of sintering. In all cases, the Na5YSi4O12-like phase (ICDD No. 32-1204) formed with no significant secondary peaks detected, indicating the purity of the synthesized N5-phase.

**Figure 4:**
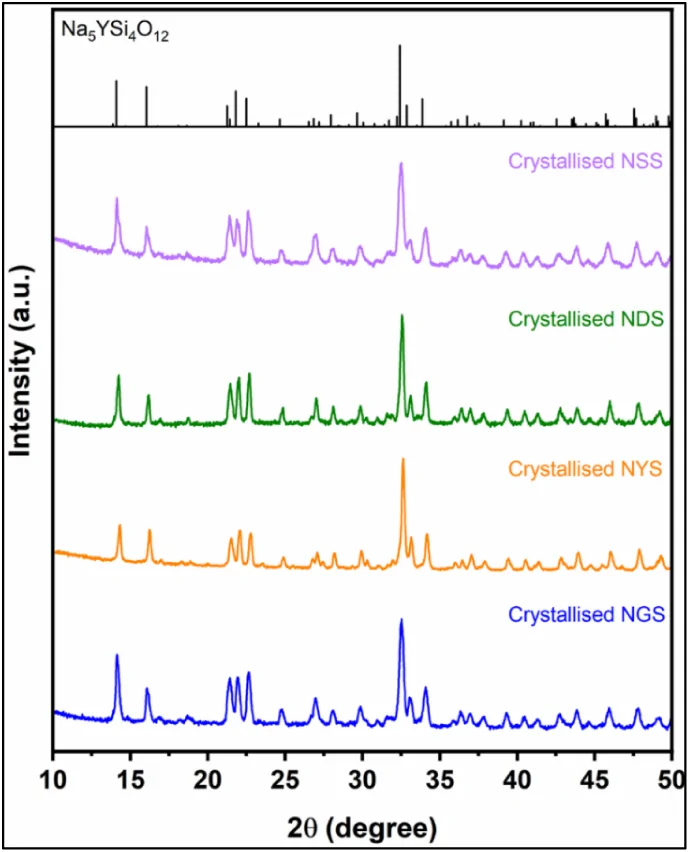
XRD patterns of NGS, NYS, NDS, and NSS glass-ceramics synthesized via melt-quench synthesis. NGS, NYS, and NDS glasses were heat-treated at 1050 °C for 4 h, while NSS was heat-treated at 950 °C for 4 h (due to the lower melting point of NSS).

The crystallization of glass to glass-ceramic was further investigated using FTIR spectroscopy. Figure 5 shows the FTIR transmittance spectra of NYS, NGS, NDS, and NSS glass and glass-ceramic samples recorded in the range 1800–400 cm^–1^. Tables S2–S5 show the FTIR band assignments for NMS glass and glass-ceramic samples. All compositions exhibit the characteristic vibrational features of silicate networks, confirming that the fundamental silicate framework is preserved during the transformation from glass to glass-ceramic.

**Figure 5:**
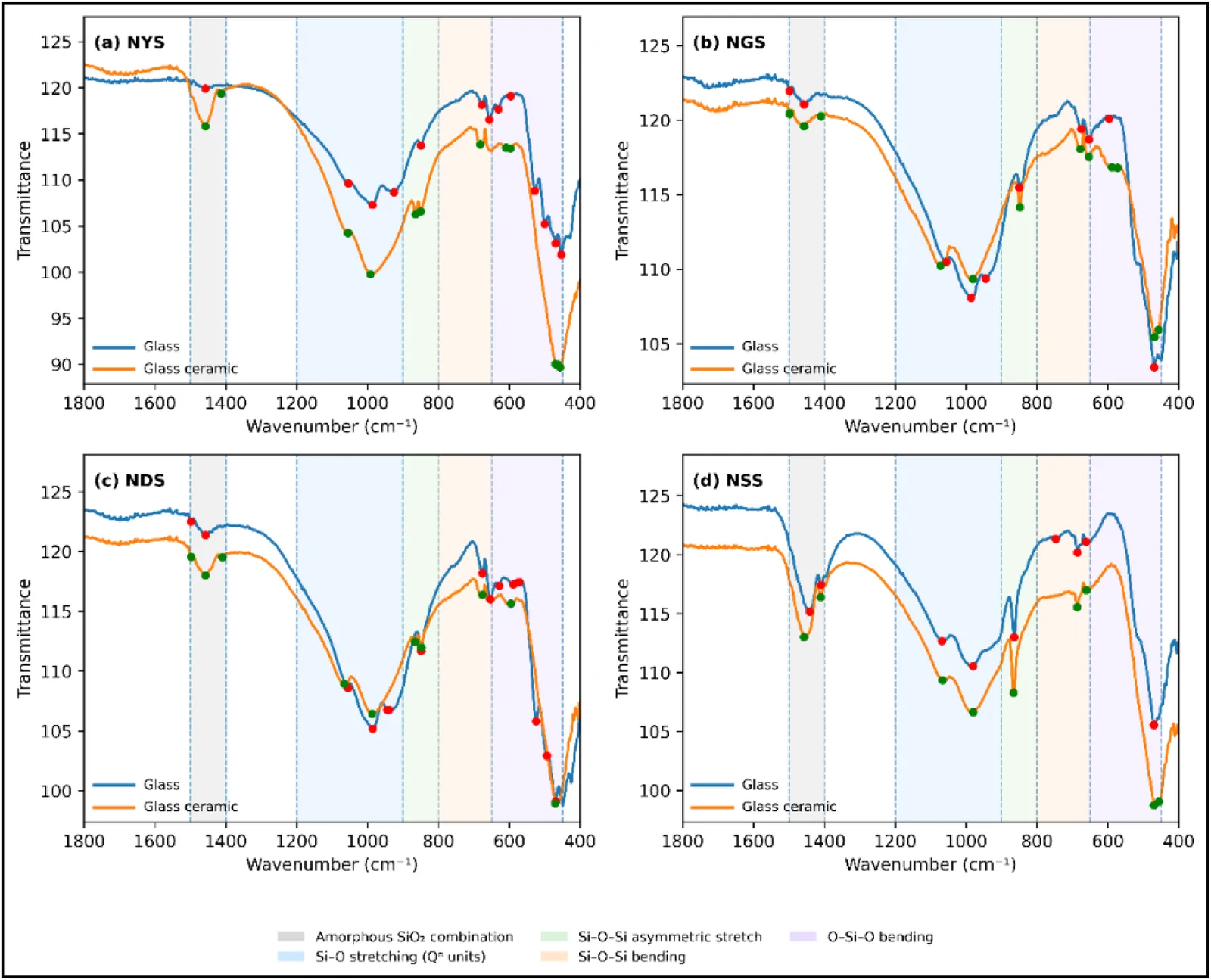
: FTIR transmittance spectra of (a) NYS, (b) NGS, (c) NDS, and (d) NSS glasses and glass-ceramics recorded in the range 1800–400 cm^–1^. Shaded regions indicate the characteristic vibrational modes of the silicate network.

The glass spectra are dominated by broad, overlapping bands, reflecting a higher degree of structural disorder, whereas the glass-ceramic spectra show sharper, better-resolved features, consistent with increased structural organization after crystallization. Weak bands observed in the 1400–1500 cm^–1^ region are attributed to combination modes involving Si–O stretching and O–Si–O bending vibrations. These bands appear in all samples and change only slightly upon crystallization, indicating that these higher-energy combination modes are relatively insensitive to the glass-to-glass-ceramic transition. The most pronounced spectral changes occur in the Si–O stretching region between 900 and 1150 cm^–1^, which is associated with Q^*n*^ silicate units and reflects the balance between bridging oxygen (BO) and nonbridging oxygen (NBO).^27^ In the glass samples, this region consists of broad, overlapping bands, suggesting the coexistence of multiple silicate environments, primarily mixed Q^3^/Q^2^ units, arising from NBOs introduced by Na^+^ modifiers. Upon crystallization, this region becomes more structured, with a reduction in band overlap and the emergence of sharper features. Rather than indicating a complete change in network connectivity, these changes point to local rearrangement and improved medium-range ordering of the silicate framework within the glass-ceramic samples. Bands in the 850–870 cm^–1^ region, assigned to asymmetric stretching vibrations of Si–O–Si linkages, exhibit slight shifts and sharpening after crystallization. This behavior reflects a narrowing of the Si–O–Si bond-angle distribution, suggesting a more constrained and uniform network connectivity. A similar trend is observed in the 650–750 cm^–1^ region, corresponding to Si–O–Si bending vibrations, where improved band definition indicates enhanced rigidity of the interconnected silicate network. In contrast, bands below 650 cm^–1^, associated with O–Si–O bending vibrations^28^ within individual silicate tetrahedra, show only small positional shifts between glass and glass-ceramic samples. This indicates that the local tetrahedral geometry remains largely unchanged, although the increased sharpness of these modes suggests greater structural stability following crystallization. Although Na–O vibrational modes are expected at low frequencies and are not clearly resolved in the present spectra, the systematic changes observed in the Si–O stretching region imply a redistribution of Na^+^ ions within the silicate framework. This interpretation is consistent with reports on Na_5_MSi_4_O_12_-type and NASICON-related glass-ceramics, in which Na^+^ ions occupy multiple coordination environments within a rigid silicate framework,^29^ thereby directly influencing ionic transport behavior.

The Raman spectra of Na_5_MSi_4_O_12_ (M = Gd, Y, Dy, Sm) glasses and corresponding glass ceramics are shown in Figure 6. Similar to other silicate glasses, the spectra can be divided into three characteristic vibrational regions: low-frequency (LF, 200–600 cm^–1^), medium-frequency (MF, 600–800 cm^–1^), and high-frequency (HF, 800–1200 cm^–1^) regions. The observed Raman bands mainly originate from vibrations of SiO_4_ tetrahedra, Si–O–Si bridging linkages, and M–O polyhedral units within the silicate network.

**Figure 6:**
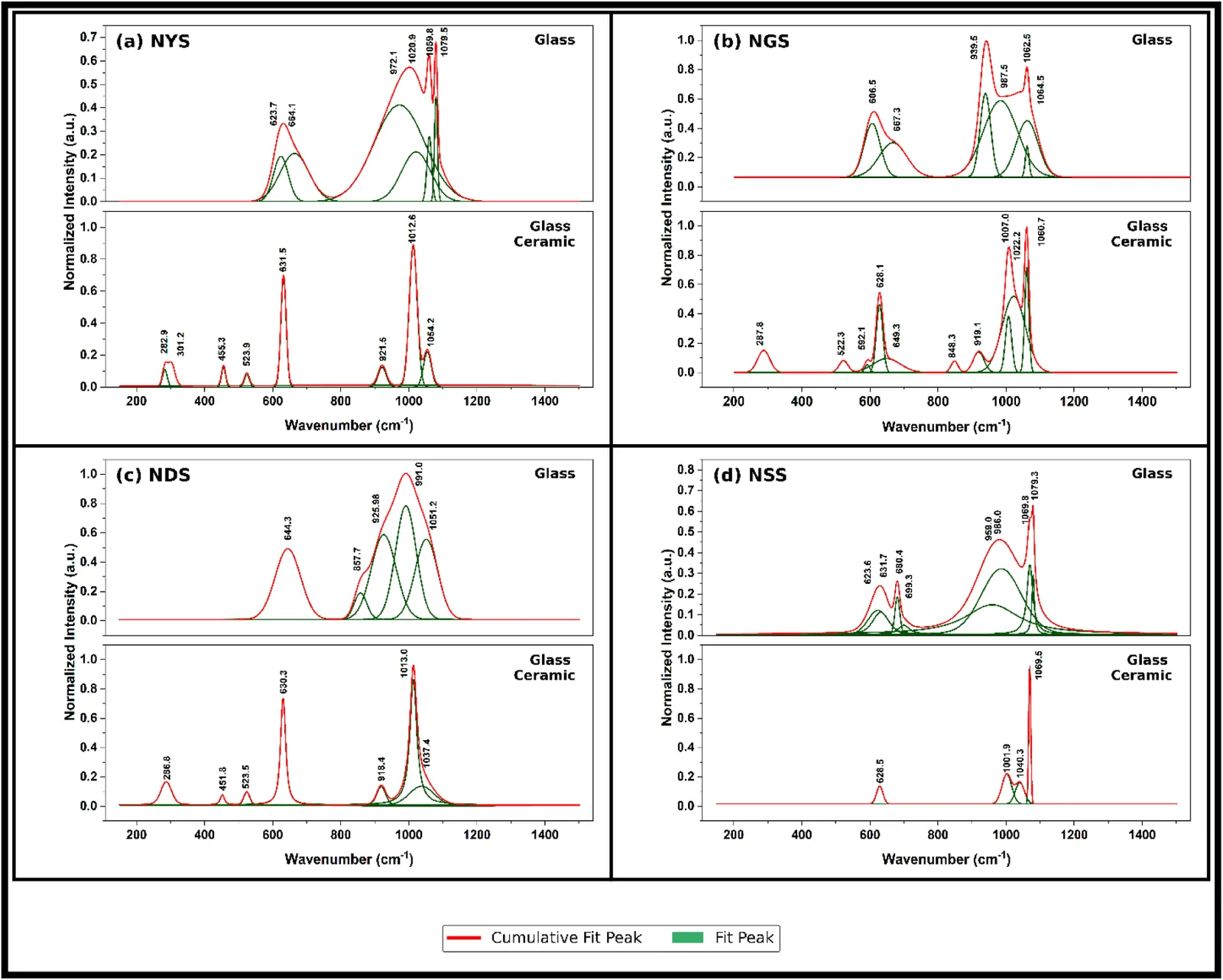
Raman spectra of (a) NYS, (b) NGS, (c) NDS, and (d) NSS glasses and glass-recorded in the range 200–1500 cm^–1^.

The Raman spectra of all glass samples exhibit broad vibrational bands (Figure 6), confirming the amorphous nature of the quenched glasses. The broadening of Raman bands arises from the random distribution of bond angles and bond lengths in the disordered glass network. In the LF region, the bands are conventionally assigned to vibrations involving bridging oxygens (BOs), O–Si–O bending modes, and vibrations associated with Na–O and rare-earth–oxygen polyhedra.

For the NYS glass sample, broad Raman bands centered near 624 and 664 cm^–1^ are observed in the MF region. These bands are attributed to symmetric stretching vibrations of Si–O–Si bridging oxygens within chain silicate structures. The intense and broad HF envelope extending from approximately 950 to 1100 cm^–1^ consists of overlapping bands located near 972, 1021, 1060, and 1079 cm^–1^. These bands are attributed to symmetric stretching vibrations of Si–O bonds involving nonbridging oxygens (NBOs), predominantly associated with Q^2^ and, to a lesser extent, with Q^3^ silicate units. The dominance of Q^2^ species indicates the formation of single-chain silicate structural units, where each SiO_4_ tetrahedron shares two bridging oxygens with neighboring tetrahedra. The asymmetry and broadness of the HF envelope suggest a distribution of Q^2^/Q^3^ environments due to structural disorder within the glass network.

However, the Raman spectrum of the NYS glass-ceramic shows significant changes, with sharp peaks at approximately 283, 301, 455, 524, 632, 921, 1013, and 1054 cm^–1^. The sharpness and enhanced intensity of these peaks confirm the formation of a highly ordered crystalline Na_5_YSi_4_O_12_ phase. The intense peaks near 1013 and 1054 cm^–1^ are assigned to Si–O stretching vibrations of ordered Q^2^ silicate tetrahedral chains, while the band near 632 cm^–1^ corresponds to Si–O–Si bridging vibrations. The low- frequency bands below 500 cm^–1^ originate from Na–O and Y–O polyhedral vibrations.

The NGS glass spectrum also shows broad LF and HF envelopes characteristic of amorphous silicate structures. The HF region contains strong bands near 939, 988, 1062, and 1064 cm^–1^, attributed to Si–O stretching vibrations of predominantly Q^2^ units with minor contributions from Q^3^ species. The relatively intense MF bands observed around 607 and 667 cm^–1^ correspond to bridging oxygen vibrations within silicate chains. However, the glass-ceramic sample exhibits sharp crystalline peaks at approximately 288, 522, 592, 628, 649, 848, 919, 1007, 1022, and 1061 cm^–1^. The sharpening of Raman modes and reduced bandwidth indicate increased short- and long-range structural ordering after crystallization. The intense peaks near 1007–1061 cm^–1^ are characteristic of ordered Si–O stretching vibrations in crystalline Na_5_GdSi_4_O_12_.

For the NDS glass sample, broad Raman bands are observed near 644, 858, 926, 991, and 1051 cm^–1^. The broad high-frequency envelope indicates the coexistence of different Q^*n*^ species, predominantly Q^2^ units with minor contributions from Q^3^ species. Following crystallization, sharp Raman peaks appear at approximately 287, 452, 524, 630, 918, 1013, and 1037 cm^–1^, confirming the development of an ordered crystalline Na_5_DySi_4_O_12_ phase. The dominant peaks near 1013 and 1037 cm^–1^ correspond to Si–O stretching modes of ordered tetrahedral chains.

Similarly, the NSS glass sample exhibits broad Raman bands around 624, 632, 680, 699, 959, 986, 1069, and 1079 cm^–1^. The strong HF envelope indicates the presence of NBO-containing predominantly Q^2^ silicate units with minor Q^3^ contributions. In the corresponding glass-ceramic sample, distinct sharp peaks are observed near 629, 1002, 1040, and 1069 cm^–1^. These sharp crystalline modes confirm the formation of ordered Na_5_SmSi_4_O_12_ glass-ceramics with enhanced tetrahedral chain regularity compared to the parent glass.

Overall, the Raman spectra clearly demonstrate the structural transformation from amorphous glass networks to highly ordered crystalline glass-ceramic phases after heat treatment. The parent glasses are dominated by disordered Q^2^ and Q^3^ silicate structural units with broad Raman envelopes arising from structural disorder and variations in local bonding environments. In contrast, the glass-ceramic samples exhibit sharp and intense Raman modes, confirming the formation of highly ordered crystalline Na_5_MSi_4_O_12_ phases. The prominent Raman bands observed within the 900–1100 cm^–1^ region are attributed to Si–O stretching vibrations of ordered single-chain silicate tetrahedra, whereas the bands near 620–680 cm^–1^ correspond to Si–O–Si bridging vibrations within silicate chains. The low-frequency modes below 550 cm^–1^ are mainly associated with Na–O and M–O polyhedral vibrations. These observations are consistent with previously reported Raman studies on Na_5_FeSi_4_O_12_.^30^

Figure 7 shows SEM micrographs of the surfaces of NMS glass ceramics. At lower magnification, all samples displayed crack-free, smooth surfaces with some roughness (possibly from sanding) and visible textured particles. It is important to heat the sample slowly and gradually to produce crack-free surfaces. The surface looked uniform and well-compacted. In the magnified view, distinct particles or crystal agglomerates are scattered across the surface of the uniform background. No pores are observed on the surfaces of the samples prepared by the MQ synthesis.

**Figure 7:**
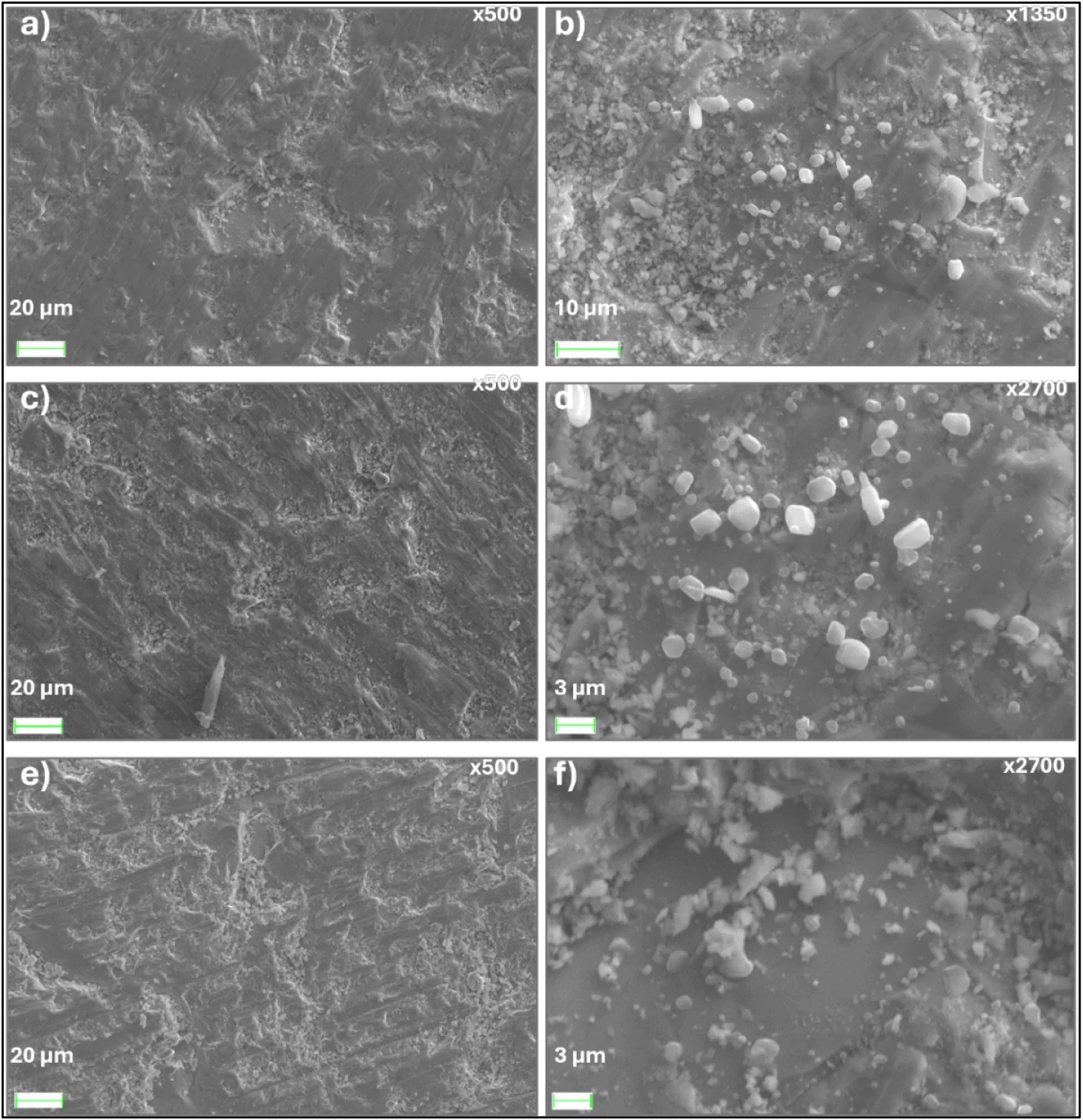
SEM micrographs of (a, b) NGS, (c, d) NYS, (e) NDS, and (f) NSS prepared by melt-quench synthesis and subsequent crystallization.

The experimental densities of NGS, NYS, NDS, and NSS ceramic samples, prepared via the MQ technique, were measured using the Archimedes method. For each sample, the average value was obtained from three measurements. NGS exhibited a density of 3.07 g cm^–3^, corresponding to 96% of its theoretical density; NYS had a density of 2.76 g cm^–3^, also representing 96% of the theoretical value; NDS demonstrated a density of 3.16 g cm^–3^, which is 97% of the theoretical density; and NSS showed a density of 3.00 g cm^–3^, corresponding to 95% of its theoretical density.

For ionic conductivity measurements, the glass pieces were shaped into regular cuboids using a glass cutter and polished with sandpaper to achieve uniform thickness. The samples were 2–3 mm thick. This preparation was necessary to facilitate ionic conductivity measurements. We analyzed the ionic conductivities of glass ceramics of NGS, NYS, NDS, and NSS using EIS. Thin Au layers were sputtered onto both sides of the shaped solid electrolyte pieces to serve as ion-blocking electrodes. Impedance measurements were conducted over a temperature range of 30–120 °C. Figure S5 presents the Nyquist plot of NGS, NYS, NDS, and NSS glass ceramics. Only a part of the semicircle for NGS and NDS, and a full semicircle for NYS and NSS, was observed in the Nyquist plot. The partially resolved semicircle in the high-to midfrequency region represents the impedance of the sample, while the polarization tail results from the accumulation of charge carriers (Na^+^ -ions) at the electrolyte/electrode interface. We were unable to separate the impedance of the grains and grain boundaries due to a depressed single semicircle. However, we calculated the total ionic conductivities and reported them in Table S6.

In the following discussion, the results for glass ceramic NMS are compared with results for NMS compounds prepared by conventional solid-state synthesis (these data will be reported in a separate publication). Glass ceramics of NGS, NYS, and NDS exhibit ionic conductivities in the range of 10^–3^ to 10^–4^ S cm^–1^ at 30 °C, while NSS exhibits slightly lower conductivity, in the range of 10^–5^ S cm^–1^ at 30 °C (Figure 8). This observation is consistent with N5 phases prepared by the solid-state route. NDS exhibited the highest conductivity in the series, with 1.24 × 10^–3^ S cm^–1^ at 30 °C, increasing to 6.39 × 10^–3^ S cm^–1^ at 120 °C. NSS exhibited the lowest conductivity among the four studied compounds, with a value of 3.30 × 10^–5^ S cm^–1^ at 30 °C, increasing to 4.07 × 10^–4^ S cm^–1^ at 120 °C. The conductivity of NGS is comparable to values reported by Schilm et al., while those for NYS and NSS are an order of magnitude lower.^22^ This paper reports NDS for the first time. The larger ionic size of Sm^3+^ would have resulted in higher ionic conductivity, but the observed lower ionic conductivity could be due to the high grain-boundary resistance of NMS, which requires further investigation.^20^

**Figure 8:**
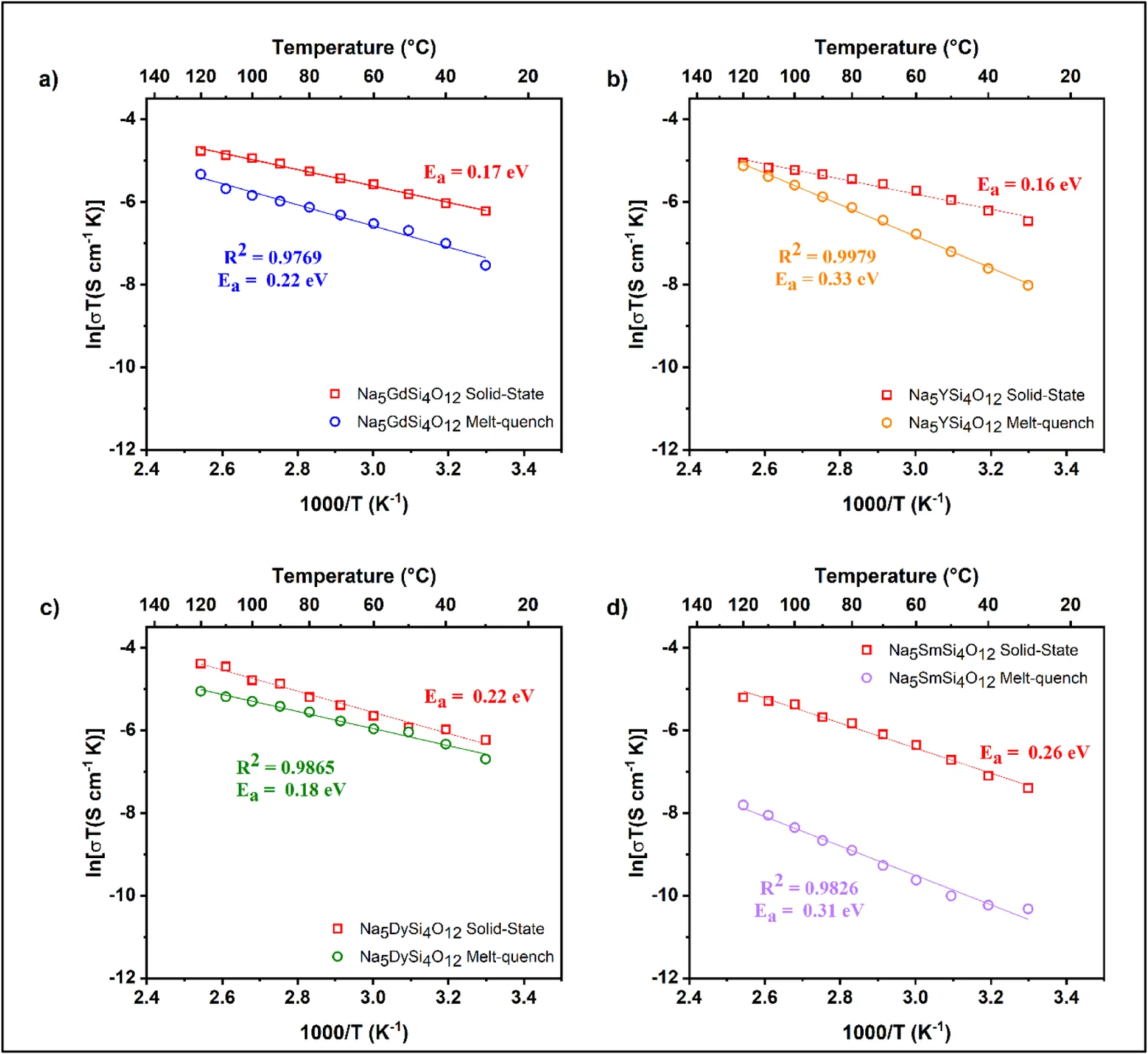
Arrhenius plots of NMS compounds prepared by solid-state route and melt-quench route: (a) NGS; (b) NYS; (c) NDS; (d) NSS.

Similar improvements in Na^+^ ionic conductivity have also been reported for other N5-type glass-ceramic electrolytes prepared by the melt-quenching and glass-crystallization route. For example, Na_5_FeSi_4_O_12_ glass-ceramics^31^ and their B-, Al-, and Ga-substituted derivatives exhibited Na^+^ ionic conductivities in the range of 1.12 × 10^–3^ to 1.69 × 10^–3^ S cm^–1^ at 300 °C, demonstrating that compositional modification within the N5 framework is an effective strategy for optimizing Na^+^ transport. Together with the present results, these studies highlight the versatility of the melt-quenching approach for developing high-performance N5-type Na^+^ glass-ceramic solid electrolytes

The order of increasing conductivity in the compounds prepared by the melt-quenching route is as follows: NSS < NYS < NGS < NDS. In NMS compounds, larger M^3+^ ions can enhance conductivity in the crystalline state by expanding the crystal lattice, thereby enabling more efficient ion migration.^32^ However, this trend is not observed for the NSS compound containing the largest Sm^3+^ ions in its structure. Full results on the temperature dependence of ionic conductivity, along with calculated ionic conductivities for samples prepared by MQ synthesis, are summarized in Table S7. Temperature-dependent EIS measurements were carried out to determine the activation energy (*E*_a_) for ion migration in the crystallized samples (Figure 8). The calculated *E*_a_ values for ion migration in the melt-quench samples are 0.22 eV for NGS, 0.33 eV for NYS, 0.18 eV for NDS, and 0.31 eV for NSS. The NGS value is lower than the previously reported value (0.26 eV), while those for NYS and NSS are higher (0.14 and 0.27 eV, respectively). The *E*_a_ value of NDS prepared by melt-quench synthesis has not been reported previously.

In general, solid electrolytes prepared via the MQ route exhibit activation energies higher than those of their SS counterparts. For NGS, the solid-state sample shows an activation energy (*E*_a_ = 0.17 eV) lower than that of the MQ sample (*E*_a_ = 0.22 eV). Similarly, NYS prepared by the SS route shows a significantly lower activation energy (*E*_a_ = 0.16 eV) compared with the MQ sample (*E*_a_ = 0.33 eV), and this could be explained by the better ionic conductivity in the sample prepared by the SS route. The NSS solid-state sample shows activation energies of 0.26 and 0.31 eV for the MQ sample. In contrast, for NDS, the activation energy is higher for the SS sample (0.22 eV) than that for the MQ sample (0.18 eV), and this difference should be further investigated. Overall, the higher activation energies observed for sodium silicate samples prepared via the MQ method can be attributed to their lower ionic conductivities, relative to those prepared by the SS route. The lower ionic conductivity and higher activation energy of glass ceramics compared to their solid-state-synthesized counterparts indicate that the sintering temperature and time need to be optimized to achieve higher conductivity.

## Conclusions

NMS-type glass ceramic solid electrolytes were successfully synthesized via melt-quenching. The thermal analysis revealed the characteristic glass transition, crystallization, and melting points of the NMS glasses, which informed the crystallization conditions for obtaining NGS, NYS, NDS, and NSS ceramics. Upon crystallization, the amorphous materials transitioned to their ceramic forms, exhibiting the N5 Phase as the primary crystalline structure, as confirmed by XRD. Among the studied compositions, NDS exhibited the highest ionic conductivity of 1.24 × 10^−3^ S cm^−1^ at 30 °C, with the lowest activation energy of 0.18 eV. In contrast, the NSS compound exhibited the lowest conductivity of 3.30 × 10^−5^ S cm^−1^ at 30 °C, while NYS exhibited the highest activation energy (0.33 eV) among the melt-quenched samples.

The synthesis method plays a pivotal role in determining the final performance of NMS solid electrolytes.^33^ Ionic conductivities of NMS compounds synthesized by conventional solid-state synthesis and by the melt-quench crystallization method reported here are compared with previously reported data obtained using the same techniques, as shown in Table S7. The relationship between the synthesis conditions and the resulting material properties is complex and remains not fully understood. Different synthesis approaches can yield variations in phase purity, grain boundary characteristics, and particle size distribution, all of which significantly affect the ionic transport. Gaining a deeper understanding of how these synthesis routes affect the structure and transport properties of NMS solid electrolytes is critical, not only for optimizing lab-scale performance but also for potential scale-up production. Even minor variations in synthesis conditions can cause substantial changes in material behavior, suggesting that ionic conductivity is not solely an intrinsic property of composition but also highly dependent on the specific synthesis method used.^33^ This highlights the need for a more refined understanding of the correlations among synthesis, microstructure, and ionic transport in solid-state electrolytes.

## Supplementary Material



## Data Availability

The data supporting this article are included in the Supporting Information.

## Notes

Digital images of glasses and glass ceramics, the Nyquist plot of NGS, EIS of glass ceramics at RT, tables on FT-IR and Raman bands assessment, a table on ionic conductivity of glass ceramics, and a comparison table on ionic conductivity values with the literature (PDF)
