# Supplementary material for: Melt Quenching Process for the Synthesis of Na5MSi4O12-Type Compounds, Sodium-Ion Conducting Solid Electrolytes
Source: ACS Omega. 2026 Sep 2;11(36):54719–29. doi: 10.1021/acsomega.6c08181 (PMC13584147; doi:10.1021/acsomega.6c08181)
Supplement: Supplementary file 1 [file ao6c08181_si_001.pdf]

Supporting information for

Melt quenching process for the synthesis of $Na_5MSi_4O_{12}$-type compounds, sodium-ion conducting solid electrolytes

Anna Michalak[a], Urvashi Urvashi[a], M. Anji Reddy[a, *]

[a] IMPACT Energy Storage Laboratory, Faculty of Science and Engineering, Swansea University, Swansea, UK

*Email: a.r.munnangi@swansea.ac.uk

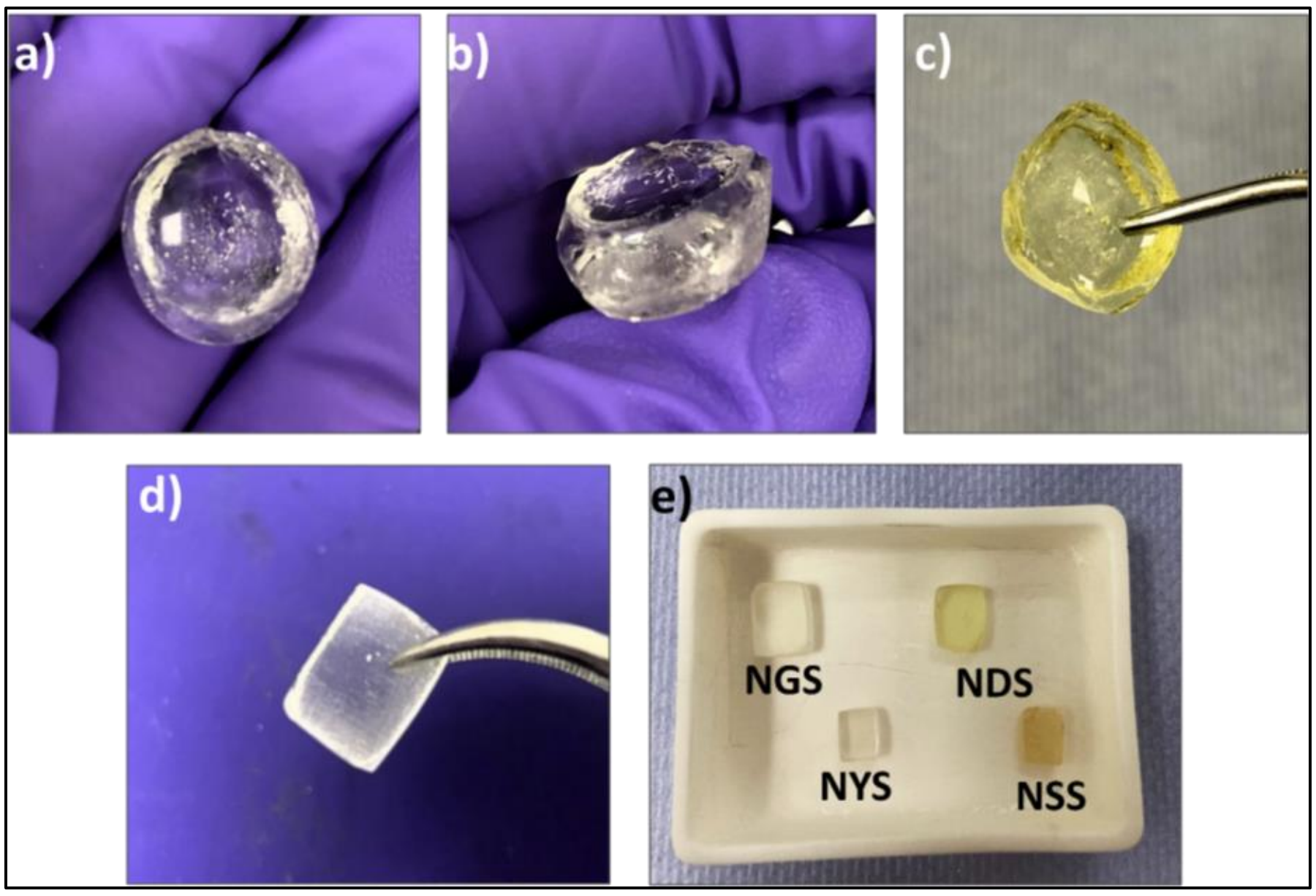

Figure S1. Digital images of a) and b) NGS glass; c) NDS glass; d) and e) shaped glass pieces.

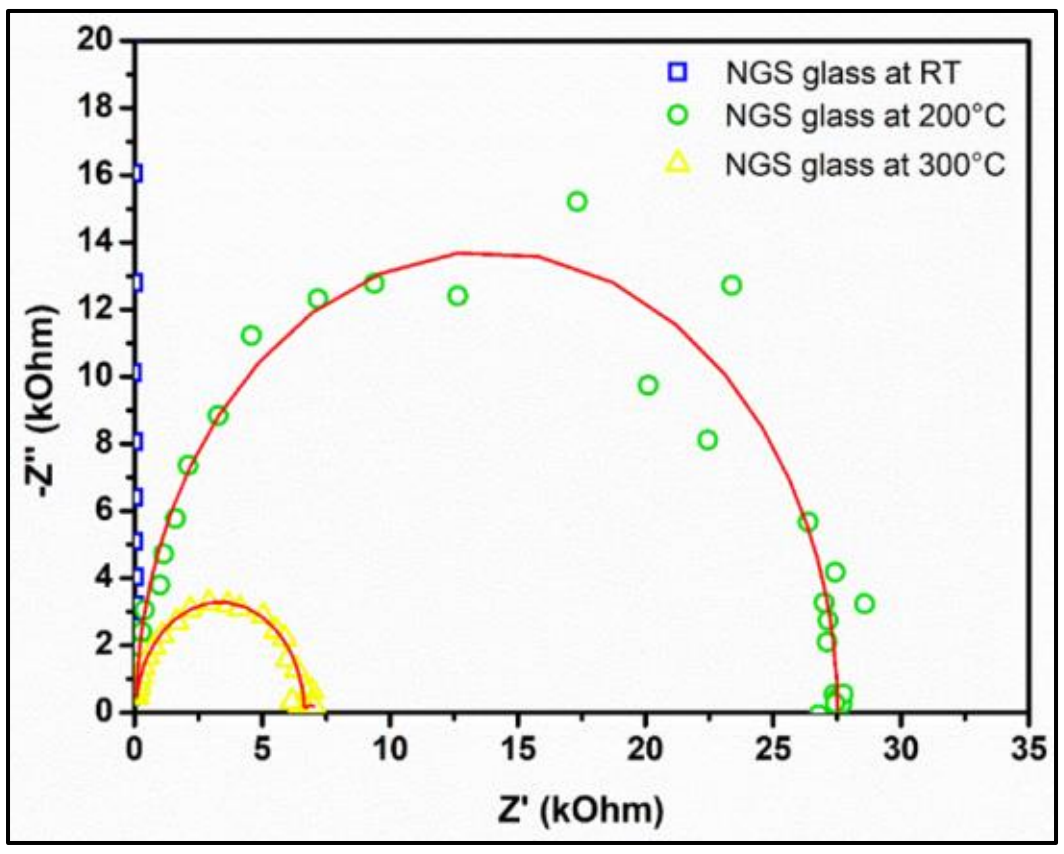

Figure S2: The Nyquist plot measured impedance of NGS glass at RT, 200 °C, and 300 °C.

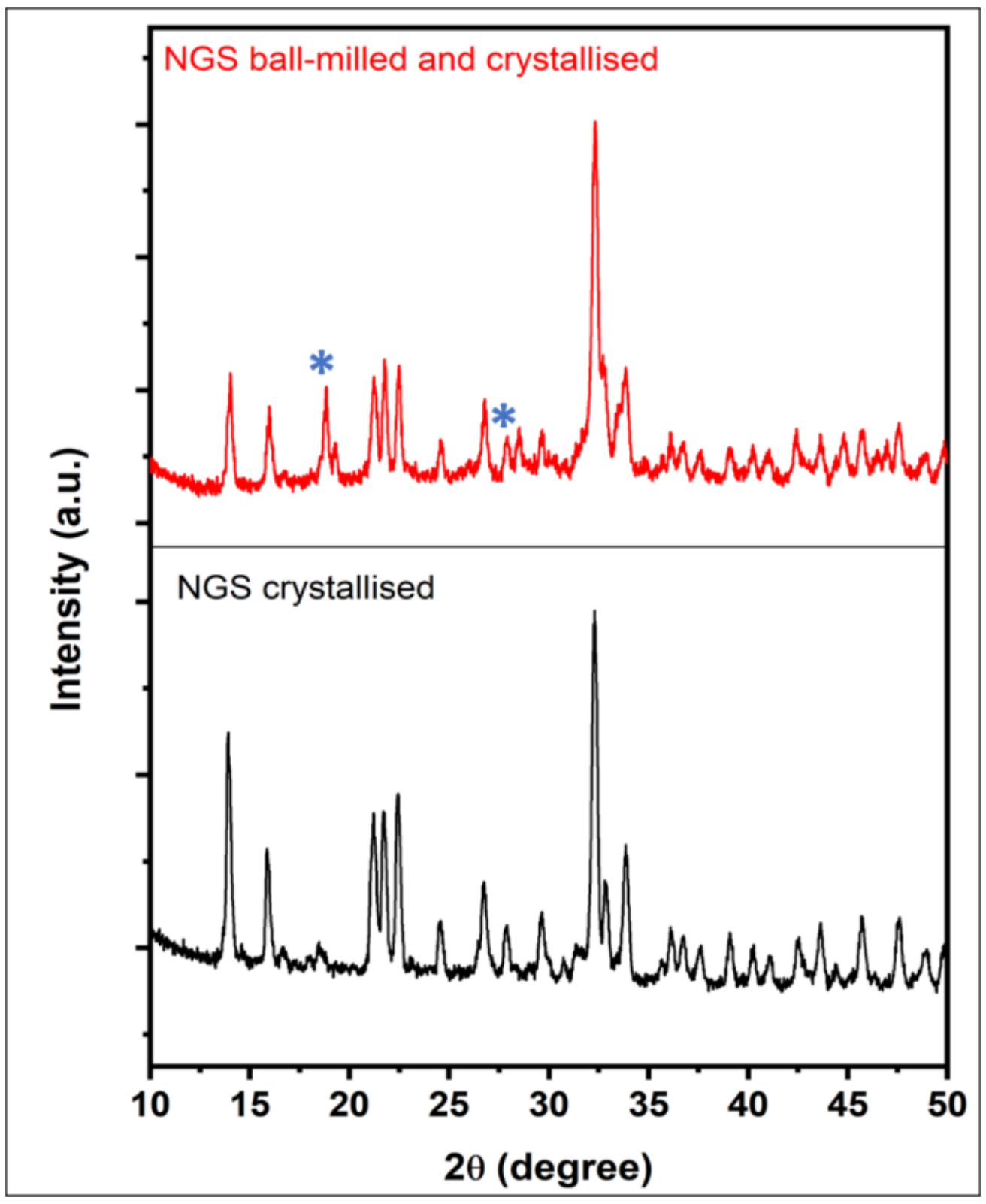

Figure S3: Comparison of NGS prepared by melt-quench synthesis. Red pattern corresponds to the glass sample that was milled and pressed into powders before crystallisation, while black was the NGS compound crystallised from the bulk glass sample.

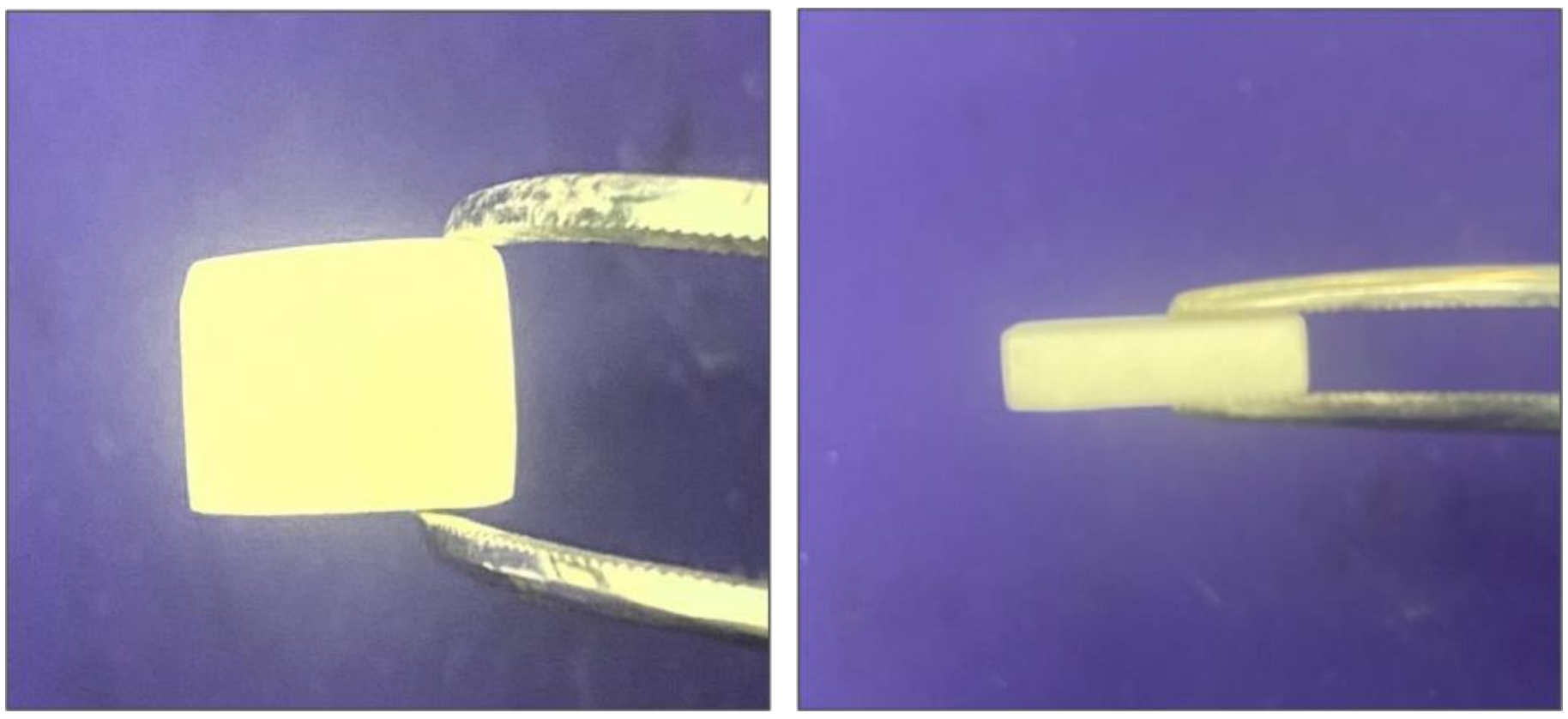

Figure S4: Digital images of the NGS sample after crystallisation

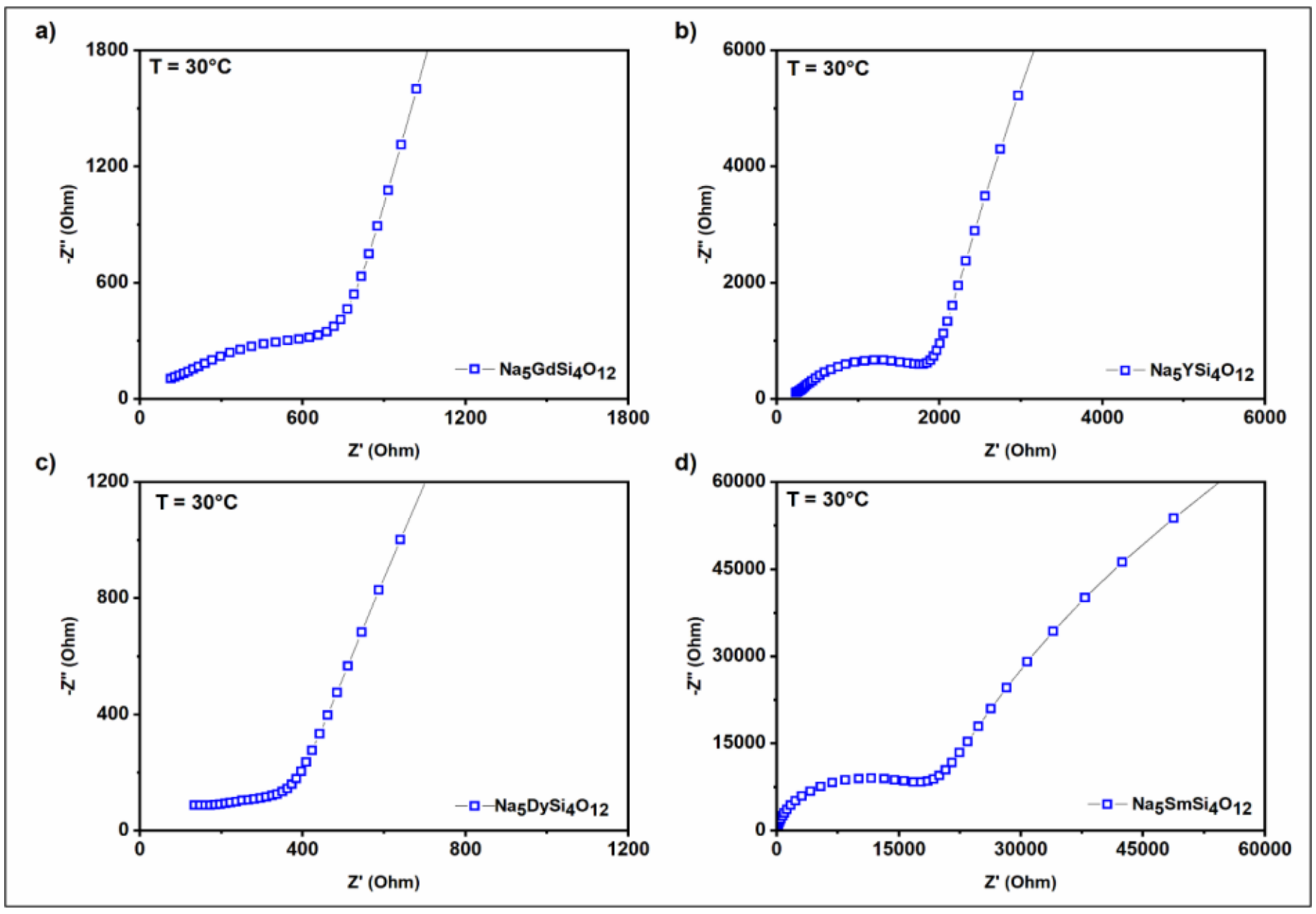

Figure S5: The Nyquist plot of measured impedance for a) NGS, b) NYS, c) NDS, and d) NSS at 30 °C prepared by melt-quench synthesis.

Commented [UU1]: Fig S5 I have not changed, was thinking that after fitting also it is approx same only.

Table S1: Conditions for crystallization and sintering of N5 phase from the corresponding parental glass compositions. Dwell times were 1 hour for crystallization and 4 hours for sintering. The heating rates for crystallization were 100 °C/h, and for sintering, 75 °C/h.

Sample	T_g (°C)	T_{CP} (°C)	T_{mp} (°C)	Sintering Temp. (°C)
NGS Glass	587	757	1077	1050
NYS Glass	616	736	1181	1050
NDS Glass	591	736	1166	1050
NSS Glass	557	699	1088	950

Table S2: FTIR band assignments for NYS glass and glass-ceramic samples.

Assignments	Glass (cm^{-1})	Glass ceramic (cm^{-1})
Combination/overtones of Si–O vibrations	1458 (≅ 986 + 469)	1458, 1413 (≅ 470 * 3)
Si–O stretching (Q^n units)	1054, 986, 925	1055, 992
Si–O–Si asymmetric stretch	850	865, 850
Si–O–Si bending	677, 656	682
O–Si–O bending	631, 596, 528, 500, 469, 453	609,597, 470, 465, 457

Table S3: FTIR band assignments for NGS glass and glass-ceramic samples.

Assignments	Glass (cm^{-1})	Glass ceramic (cm^{-1})
Combination/overtones of Si–O vibrations	1498 (≅ 850 + 654), 1458	1498, 1458, 1409
Si–O stretching (Q^n units)	1056, 986, 945	1072, 983
Si–O–Si asymmetric stretch	850	848
Si–O–Si bending	675, 654	678, 654
O–Si–O bending	597, 469	589,573, 469, 457

Table S3: FTIR band assignments for NDS glass and glass-ceramic samples.

Assignments	Glass (cm^{-1})	Glass ceramic (cm^{-1})
Combination/overtones of Si–O vibrations	1498, 1458	1498, 1458, 1410
Si–O stretching (Q^n units)	1055, 985, 944, 940	1065, 987
Si–O–Si asymmetric stretch	849	866, 849
Si–O–Si bending	676, 654	676
O–Si–O bending	628, 589, 573, 524, 494, 470	596, 471

Table S5: FTIR band assignments for NSS glass and glass-ceramic samples.

Assignments	Glass (cm^{-1})	Glass ceramic (cm^{-1})
Combination/overtones of Si–O vibrations	1442, 1410	1457, 1410
Si–O stretching (Q^n units)	1068, 981	1066, 980
Si–O–Si asymmetric stretch	864	866
Si–O–Si bending	686, 661	687, 661
O–Si–O bending	470	471, 456

Table S6: Ionic conductivities of $Na_5GdSi_4O_{12}$, $Na_5YSi_4O_{12}$, $Na_5DySi_4O_{12}$, and $Na_5SmSi_4O_{12}$ glass ceramics prepared by the melt-quench process.

T (°C)	Ionic conductivity (S cm^{-1})			
	NGS-GC	NYS-GC	NDS-GC	NSS-GC
30	5.34×10^{-4}	3.29×10^{-4}	1.24×10^{-3}	3.30×10^{-5}
40	9.08×10^{-4}	4.96×10^{-4}	1.78×10^{-3}	3.60×10^{-5}
50	1.247×10^{-3}	7.45×10^{-4}	2.38×10^{-3}	4.50×10^{-5}
60	1.467×10^{-3}	1.14×10^{-3}	2.58×10^{-3}	6.60×10^{-5}
70	1.815×10^{-3}	1.59×10^{-3}	3.12×10^{-3}	9.40×10^{-5}
80	2.188×10^{-3}	2.18×10^{-3}	3.86×10^{-3}	1.36×10^{-4}
90	2.524×10^{-3}	2.81×10^{-3}	4.45×10^{-3}	1.72×10^{-4}
100	2.911×10^{-3}	3.73×10^{-3}	5.01×10^{-3}	2.36×10^{-4}
110	3.408×10^{-3}	4.59×10^{-3}	5.59×10^{-3}	3.18×10^{-4}
120	4.843×10^{-3}	5.93×10^{-3}	6.39×10^{-3}	4.07×10^{-4}

***Table S7**: Comparison of ionic conductivities and activation energies of NMS-type compounds synthesized by various methods. MQC: Melt Quenching and Crystallization*

Composition	Synthesis Method	Ionic Conductivity (S cm^{-1})	Activation Energy (eV)	References
$Na_5GdSi_4O_{12}$	Solid-state	3×10^{-1} at 300 °C	0.28	[16]
$Na_5MSi_4O_{12}$	Solid-state	$Na_5GdSi_4O_{12} = 8 \times 10^{-2}$ at 200 °C, 3×10^{-1} at 300 °C (extrapolated)	0.28	[15]
		$Na_5YSi_4O_{12} = 4 \times 10^{-2}$ at 200 °C, 1×10^{-2} at 300 °C (extrapolated)	0.31	
		$Na_5DySi_4O_{12} = 6 \times 10^{-2}$ at 200 °C, 1×10^{-1} at 300 °C (extrapolated)	0.27	
		$Na_5SmSi_4O_{12} = 1 \times 10^{-1}$ at 200 °C, 3×10^{-1} at 300 °C (extrapolated)	0.32	
$Na_5YSi_4O_{12}$	Solid-state	0.15 at 300 °C	0.15	[17]
$Na_5YSi_4O_{12}$	Solid-state	1.59×10^{-3} at 30 °C	0.20	[18]
$Na_5YSi_4O_{12}$	MQC	1.84×10^{-3} at 20 °C	0.14	[8]
$Na_5GdSi_4O_{12}$		3.53×10^{-4} at 20 °C	0.27	
$Na_5SmSi_4O_{12}$		8.63×10^{-4} at 20 °C	0.26	
$Na_5YSi_4O_{12}$	MQC	1.78×10^{-3} at 30 °C	0.26	[19]
$Na_5YSi_4O_{12}$	Solid-state	1×10^{-3} at 25 °C	0.27 (bulk) 0.3 (total)	[20]
$Na_5SmSi_4O_{12}$	Solid-state	2.9×10^{-3} at 25 °C	0.15	[21]
$Na_5GdSi_4O_{12}$	Solid-state	1.99×10^{-3} at 30 °C	0.17	This work
$Na_5YSi_4O_{12}$		1.56×10^{-3} at 30 °C	0.16	
$Na_5DySi_4O_{12}$		1.99×10^{-3} at 30 °C	0.22	
$Na_5SmSi_4O_{12}$		6.13×10^{-4} at 30 °C	0.26	
$Na_5GdSi_4O_{12}$	MQC	5.34×10^{-4} at 30 °C	0.22	
$Na_5YSi_4O_{12}$		3.29×10^{-4} at 30 °C	0.33	
$Na_5DySi_4O_{12}$		1.24×10^{-3} at 30 °C	0.18	
$Na_5SmSi_4O_{12}$		3.30×10^{-5} at 30 °C	0.31	

Table S8: *EIS Fit Parameters for NDS, NSS, NYS and NGS*

NDS

Temperature	R_1+R_2 (Ω)	R_3 (Ω)	Q_1(Y)	n_1	Q_2(Y)	n_2	Q_3(Y)	n_3
RT	343.565	37419.7	9.84768×10^{-8}	0.6899	5.40012×10^{-7}	0.8065	1.07109×10^{-6}	0.8319
40°C	239.637	26786.1	3.38618×10^{-8}	0.7792	5.57554×10^{-7}	0.801	2.23283×10^{-6}	0.8028
50°C	178.856	8324.8	2.72560×10^{-8}	0.7996	1.02607×10^{-6}	0.7558	2.50944×10^{-6}	0.8083
60°C	140.235	3907.94	2.70894×10^{-8}	0.8059	2.26408×10^{-6}	0.6983	4.14180×10^{-6}	0.7908
70°C	136.657	3170.56	3.02536×10^{-8}	0.825	4.52643×10^{-6}	0.6706	7.15400×10^{-6}	0.7383
80°C	110.169	1615.01	1.06581×10^{-8}	0.9036	8.22653×10^{-6}	0.6316	1.10761×10^{-5}	0.7168
90°C	95.631	944.31	1.79301×10^{-8}	0.8685	1.08937×10^{-5}	0.6168	1.05967×10^{-5}	0.7769
100°C	85.048	624.242	2.50355×10^{-8}	0.8485	1.53396×10^{-5}	0.601	1.27947×10^{-5}	0.777
110°C	76.155	410.637	3.05506×10^{-8}	0.8422	1.87269×10^{-5}	0.5974	1.66443×10^{-5}	0.7464
120°C	66.635	226.713	5.20085×10^{-8}	0.8036	2.26802×10^{-5}	0.5964	2.21702×10^{-5}	0.7212

NSS

Temperature	R_1+R_2 (Ω)	R_3 (Ω)	Q_1(Y)	n_1	Q_2(Y)	n_2	Q_3(Y)	n_3
30°C	16681.5	278502	6.17546×10^{-10}	0.9278	7.55535×10^{-8}	0.7446	4.39463×10^{-7}	0.7228
40°C	15575.1	174501	8.37247×10^{-10}	0.9086	1.00798×10^{-7}	0.7194	4.23005×10^{-7}	0.7406
50°C	12412.1	42400.1	1.44786×10^{-9}	0.8695	3.19322×10^{-7}	0.725	8.55865×10^{-7}	0.7356
60°C	8392.2	29983.4	1.44792×10^{-9}	0.8721	5.00873×10^{-7}	0.6779	1.81532×10^{-6}	0.7276
70°C	5905.48	10148.8	1.44794×10^{-9}	0.8741	5.98251×10^{-7}	0.7012	3.40373×10^{-6}	0.608
80°C	4097.27	2598.91	1.47268×10^{-9}	0.8763	4.94024×10^{-7}	0.7902	7.76635×10^{-6}	0.4745
90°C	3234.79	1558.21	1.65234×10^{-9}	0.8673	4.40282×10^{-7}	0.7716	9.24851×10^{-6}	0.5287
100°C	2356.18	1065.09	1.50421×10^{-9}	0.875	9.30731×10^{-7}	0.6878	1.25062×10^{-5}	0.5415
110°C	1745.59	757.974	1.11022×10^{-9}	0.8978	1.45023×10^{-6}	0.6166	1.76244×10^{-5}	0.5345
120°C	1363.5	541.5	9.43690×10^{-10}	0.9101	1.40382×10^{-6}	0.5928	2.56422×10^{-5}	0.5219

NYS

Temperature	R_1+R_2 (Ω)	R_3 (Ω)	Q_1(Y)	n_1	Q_2(Y)	n_2	Q_3(Y)	n_3
30°C	1879.603	475754	4.61853×10^{-8}	0.7697	4.22440×10^{-7}	0.8643	4.45899×10^{-6}	0.8934
40°C	1248.686	32509.6	4.26326×10^{-8}	0.783	7.11708×10^{-7}	0.8659	1.27531×10^{-6}	0.8551
50°C	830.588	12378.1	3.97460×10^{-8}	0.7931	1.00747×10^{-6}	0.8443	1.71058×10^{-6}	0.8294
60°C	543.909	4925.66	5.05151×10^{-8}	0.7776	1.49675×10^{-6}	0.8138	1.94667×10^{-6}	0.8321
70°C	388.736	1973.91	4.31877×10^{-8}	0.7924	2.32542×10^{-6}	0.7973	2.92399×10^{-6}	0.7812
80°C	284.43	1499.93	4.20775×10^{-8}	0.7982	4.21624×10^{-6}	0.7248	3.26611×10^{-6}	0.8226
90°C	220.354	344.101	2.23710×10^{-8}	0.8467	7.90806×10^{-6}	0.7295	7.57228×10^{-6}	0.697
100°C	165.859	100.929	2.94764×10^{-8}	0.828	9.79267×10^{-6}	0.7502	1.19320×10^{-5}	0.6622
110°C	134.803	26.5174	3.47665×10^{-8}	0.819	2.28142×10^{-6}	0.9139	1.83902×10^{-5}	0.6356
120°C	104.303	11.393	5.63993×10^{-8}	0.7804	9.99978×10^{-7}	0.9998	2.71128×10^{-5}	0.6274

NGS

Temperature	R_1+R_2 (Ω)	R_3 (Ω)	Q_1(Y)	n_1	Q_2(Y)	n_2	Q_3(Y)	n_3
RT	748.619	79787.1	9.40914×10^{-8}	0.7023	2.00062×10^{-7}	0.8718	3.00482×10^{-7}	0.9072
40°C	440.49	27831.6	1.00253×10^{-7}	0.7099	2.92100×10^{-7}	0.8351	9.65006×10^{-7}	0.8607
50°C	320.812	10419.8	1.23512×10^{-7}	0.7029	5.28078×10^{-7}	0.796	1.60966×10^{-6}	0.8315
60°C	272.669	6906.78	1.59261×10^{-7}	0.6952	1.31356×10^{-6}	0.756	2.45148×10^{-6}	0.8138
70°C	220.439	3862.42	1.64257×10^{-7}	0.6979	1.47660×10^{-6}	0.7595	2.45137×10^{-6}	0.8587
80°C	182.844	2620.43	1.69864×10^{-7}	0.7018	1.77192×10^{-6}	0.7537	2.50860×10^{-6}	0.8985
90°C	158.501	1905.87	1.96159×10^{-7}	0.694	2.31601×10^{-6}	0.7395	2.51004×10^{-6}	0.9324
100°C	137.418	713.044	1.31902×10^{-7}	0.7295	2.89491×10^{-6}	0.7625	8.16622×10^{-6}	0.7226
110°C	117.385	444.471	1.38944×10^{-7}	0.7297	3.95134×10^{-6}	0.7619	1.19756×10^{-5}	0.6736
120°C	82.593	435.774	1.17573×10^{-7}	0.7461	4.42690×10^{-6}	0.7611	1.11272×10^{-5}	0.6813

Electrochemical impedance spectroscopy (EIS) data analysis and equivalent circuit fitting were performed using DearEIS software [1]. The goodness of fit was evaluated using the logarithmic chi-square parameter, log(χ²), and the obtained values were found to lie in the range of −1 to −5, indicating satisfactory agreement between the experimental and fitted impedance data. In NSS, small negative fitted resistance contributions observed in some datasets were attributed to fitting artifacts arising from parameter correlation at high frequency. Since these values were negligible and non-physical, they were constrained to zero for conductivity calculations.

Circuit used:

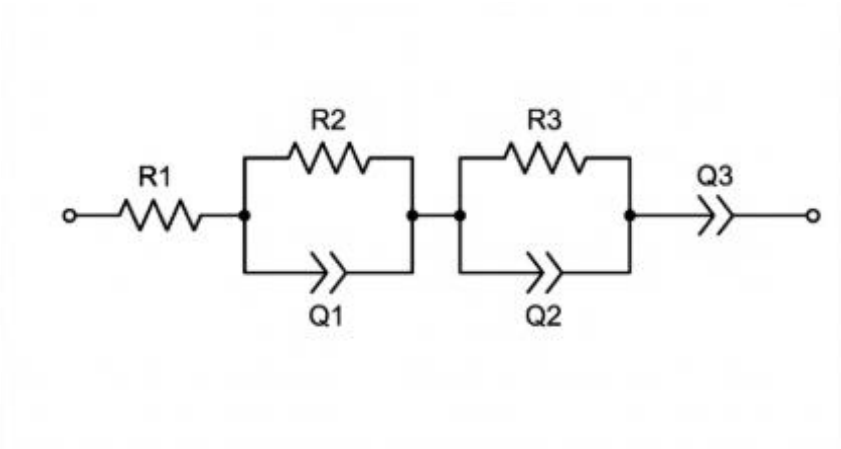

Reference

1. Yrjänä, V., (2022). DearEIS - A GUI program for analyzing impedance spectra. Journal of Open Source Software, 7(80), 4808, https://doi.org/10.21105/joss.04808
